# Recruitment to publicly funded trials — Are surgical trials really different?

**DOI:** 10.1016/j.cct.2008.02.005

**Published:** 2008-09

**Authors:** Jonathan A. Cook, Craig R. Ramsay, John Norrie

**Affiliations:** a Health Services Research Unit, University Of Aberdeen, Aberdeen, UK; b Centre for Health Care Randomised Trials, University Of Aberdeen, Aberdeen, UK

**Keywords:** Randomised controlled trials, Surgery, Recruitment, Publicly funded

## Abstract

**Background:**

Good recruitment is integral to the conduct of a high-quality randomised controlled trial. It has been suggested that recruitment is particularly difficult for evaluations of surgical interventions, a field in which there is a dearth of evidence from randomised comparisons. While there is anecdotal speculation to support the inference that recruitment to surgical trials is more challenging than for medical trials we are unaware of any formal assessment of this. In this paper, we compare recruitment to surgical and medical trials using a cohort of publicly funded trials.

**Data:**

Overall recruitment to trials was assessed using of a cohort of publicly funded trials (*n* = 114). Comparisons were made by using the Recruitment Index, a simple measure of recruitment activity for multicentre randomised controlled trials. Recruitment at the centre level was also investigated through three example surgical trials.

**Results:**

The Recruitment Index was found to be higher, though not statistically significantly, in the surgical group (*n* = 18, median = 38.0 IQR (10.7, 77.4)) versus (*n* = 81, median = 34.8 IQR (11.7, 98.0)) days per recruit for the medical group (median difference 1.7 (− 19.2, 25.1); *p* = 0.828). For the trials where the comparison was between a surgical and a medical intervention, the Recruitment Index was substantially higher (*n* = 6, 68.3 (23.5, 294.8)) versus (*n* = 93, 34.6 (11.7, 90.0); median difference 25.9 (− 35.5, 221.8); *p* = 0.291) for the other trials.

**Conclusions:**

There was no clear evidence that surgical trials differ from medical trials in terms of recruitment activity. There was, however, support for the inference that medical versus surgical trials are more difficult to recruit to. Formal exploration of the recruitment data through a modelling approach may go some way to tease out where important differences exist.

## 1 Background

Good recruitment is integral to the conduct of a high-quality randomised controlled trial. However, many trials struggle to recruit to their original target in terms of both time and budget [1–3]. It has been suggested that recruitment is particularly difficult for evaluations of surgical interventions, a field in which there is a dearth of evidence from randomised comparisons [4–6]. Strong preferences amongst surgeons and potential participants, as well as limitations on resources both infrastructure (theatre time) and staff availability (surgical team), have been highlighted as possible explanations.

A surgical trial can be defined as a trial undertaking a randomised comparison of a surgical procedure against some form of control. Various options are available for the control, including where viable, a surgical placebo. In general, surgical trials can be considered to fall into three levels of increasing difficulty to conduct:

• Level 1 — randomised comparisons of surgical procedures which differ only in a minor way (e.g. a comparison of two methods of suturing) [7]
• Level 2 — randomised comparisons of different forms of surgery which differ in a significant way in terms of the overall approach and skills required (e.g. a comparison of laparoscopic surgery versus open surgery for inguinal hernia) [8]
• Level 3 — randomised comparisons of some form of medical management versus a surgical intervention (e.g. proton pump inhibitors versus fundoplication for gastro-oesophageal reflux disease) [9].

Issues of patient preference and clinical equipoise are likely to be greatest where the difference between comparisons is greatest [10].

While there is anecdotal speculation to support the inference that recruitment to surgical trials is more challenging than for medical trials, we are unaware of any formal assessment of this. The aim of this work was to use empirical data to assess whether there is evidence that recruitment to surgical trials is more difficult than for medical trials. Two aspects were considered, overall recruitment to the trial (trial level) through the assessment of a cohort of trials, and recruitment at the centre level through three example surgical trials.

## 2 Methods

We assessed recruitment to surgical trials in general by testing two pre-specified hypotheses on data from a cohort of publicly funded trials. We firstly hypothesised that surgical trials would be more difficult to recruit to and therefore they have a higher level of recruitment activity than medical trials (Hypothesis A) and secondly that the surgical trials of greater complexity (as defined earlier) would similarly lead to a higher level of recruitment activity (Hypothesis B).

To test Hypotheses A and B, we used a simple measure of recruitment activity for multicentre randomised controlled trials, the Recruitment Index [11], which is defined as the average number of days taken to recruit a participant in a centre:

$$RI=\frac{RecruitmentPeriod\times No.centres}{No.participants}\text{.}$$

For example, a trial which recruited 100 patients in 200 days over 5 centres would have a RI of 10 (average number of centre recruitment days per recruit). We calculated the RI for a cohort of publicly funded trials to measure the level of recruit activity for surgical trials. The RI was calculated on the basis of the actual number recruited as opposed to the number completing the trial protocol which was not consistently recorded. We tested for an overall difference between surgical and medical trials to assess Hypothesis A. Level 3 trials versus the remaining trials was tested to assess Hypothesis B.

For hypothesis generating purposes, and to illustrate complexities of multicentre recruitment within surgical trials, two hypotheses were tested on three example surgical trials. First, we hypothesised that late starting centres would recruit less than early starting centres (Hypothesis C). Second, we hypothesised that the rate of recruitment within centres would reduce during the course of the trial (Hypothesis D).

All hypotheses were tested using a Mann–Whitney *U* test at the 5% significant level in SPSS [12]. The Recruitment Index, the number recruited and the rate of recruitment were summarised as median and interquartile range (IQR). A 95% confidence interval for the median difference was calculated in STATA [13].

## 3 Data

### 3.1 Cohort

The STEPS project carried out a review of publicly funded trials from two UK funding bodies, the UK NHS R&D National Methodology Programme and the UK Medical Research Council (MRC) [2]. All multicentre trials except for cluster randomised trials were included. Data was collected on the 114 multicentre trials, which recruited between 1994 and 2002, on recruitment and finance details from application forms and progress reports. Where insufficient data was available in the STEPS database a search for trial publications was undertaken to collect additional details which may not have been available when the original search was conducted. For individual trials recruited to more than one randomised comparison, only the comparison with the largest target recruitment was considered. Though UK funded, 25 (22%) of the trials also had centres based outside the UK. A pilot study had been undertaken for 60 of the Trials (53%). Two reviewers independently categorised the cohort as either a surgical or medical trial (Table 1). Any differences were resolved by consensus.

### 3.2 Example trials

Centre level recruitment data was available for three surgical trials, one representing each of the three levels of surgical trials. Basic information on the three surgical trials used to assess multicentre recruitment is given in Table 2. The trials differed in size and clinical area.

## 4 Results

### 4.1 Cohort

The cohort included trials from a variety of clinical areas (including Cancer, HIV/AID, urology, primary care and mental health) and setting (hospital, community and general practice). Trial varied greatly in their target recruitment between 60 and 66,000. The number of centres, number of participants recruited and the time recruiting (start of recruitment to end of recruitment) are given in Table 1 for surgical and medical trials.

The Recruitment Index was found to be higher, though not statistically significantly, in the surgical group (*n* = 18, median = 38.0 IQR (10.7, 77.4)) versus (*n* = 81, median = 34.8 IQR (11.7, 98.0)) days per recruit for the medical group (median difference 1.7 (− 19.2, 25.1); *p* = 0.828). For trials which compared a surgical against a medical comparison (level 3), the Recruitment Index was substantially higher (*n* = 6, 68.3 (23.5, 294.8)) versus (*n* = 93, 34.6 (11.7, 90.0); median difference 25.9 (− 35.5, 221.8); *p* = 0.291) for the other trials.

### 4.2 Example trials

The results for Hypotheses C and D are given in Tables 3 and 4 respectively. Two of the three trials supported Hypothesis C that latter centres do not recruit as well as early centres. The median rate of recruitment for latter centres was approximately a half that of the early centres for trials 1 and 2.

All 3 tended towards a reduction in the rate of recruitment within centre (Hypothesis D) with one significant at the 5% level and one just failing to be so. Substantial reductions in the number of participants recruited were observed between the first half of centre's recruitment period and the second half.

## 5 Discussion

In general, there was no clear evidence that surgical trials differ from medical trials in terms of recruitment activity. There was, however, support for the inference that complex (level 3) surgical trials are more difficult to recruit to. We suggest that the 3 levels of surgical trials is a useful paradigm for understanding the variation in required recruitment activity between surgical trials.

The Recruitment Index is a simple measure of the recruitment rate for a multicentre trial. We found that the index varied greatly between trials and it might be the case that a more nuanced measure may be more sensitive to differences between trials. For example, extending the measure to the centre level might provide a more accurate picture of recruitment as centres may have staggered start dates.

The exploratory centre level analysis illustrated that recruitment is a complex process within a trial and emphasised the variation in recruitment both between and within centres. Haidich and Ioannidis [14] previously demonstrated a difference between late starting centres and early centres for AIDS trials and we found a broadly consistent pattern. There was also some evidence of slowing down in recruitment during the trial period.

Patient preference is often the most notable reason for recruitment being difficult to surgical trials [15]. Surgical treatment polarises participant attitudes for and against surgery. Further research is needed to investigate whether other factors also play a part and to what degree recruitment strategies can improve participation rates for these trials.

There were a number of limitations to our study. To enable calculation of the Recruitment Index we used the number of participant randomised. This ignored the quality of data on those included in the trial. The need for long-term follow up to evaluate surgical interventions has been highlighted and consideration of retainment of participant was not assessed [5]. Though we looked at a large cohort of trials there was a relatively small number of surgical trials available. Similarly, we only considered three surgical trials at the centre level and therefore cautious interpretation is needed. It is uncertain whether our results would hold for commercial as opposed to publicly funded trials and investigation of this is warranted.

### 5.1 Conclusions

We found no clear evidence to support the assertion that recruitment to surgical trials in general is more difficult than other clinical areas. However, complex (medical versus surgical) trials appear substantially more difficult to recruit to. Formal exploration of the recruitment data through a modelling approach may go some way to tease out where important differences exist and could inform future trial design.

## Funding

The first author was supported by a Medical Research Council UK Fellowship.

## Figures and Tables

**Table 1:** Summary recruitment information on the cohort of multicentre trials

Trial feature — median (IQR)	Surgical trials	Medical trials
	*N* = 18	*N* = 81
Number of centres	12 (4, 28)	16 (5, 49)
Number recruited	449 (204, 915)	368 (225, 872)
Time recruiting (days)	1162 (730, 1472)	912 (649, 1272)

**Table 2:** Example surgical trials summary information

Surgical trial level	Clinical area	No. of centres	Actual (target) recruitment	RI
1	Orthopaedics	27	1715 (1500)	20.4
2	General Surgery	26	1027 (1000)	30.9
3	Gastroenterology	20	357 (600)	67.6

**Table 3:** Number of participants recruited in early and late starting centres for 3 example surgical trials

Example trial	Number of participants recruited per month — median (IQR)				*p* value
Early centres		Late centres	
*n*	Median (IQR)	*n*	Median (IQR)
1	13	4.1 (2.6, 5.0)	14	1.8 (1.4, 4.8)	0.077
2	13	1.9 (1.0, 2.7)	13	1.0 (0.7, 1.7)	0.065
3	10	0.8 (0.5, 1.2)	10	0.9 (0.8, 1.9)	0.369

**Table 4:** Number of participant recruited within centres for 3 example surgical trials

Example trial	Number of participants recruited — median (IQR)		*p* value
1st half of recruitment period	2nd half of recruitment period
1	30 (11, 44)	21 (6, 45)	0.052
2	8 (4, 19)	6 (2, 23)	0.122
3	10 (6, 14)	5 (1, 11)	0.022

## References

[1] Lovato L.C., Hill K., Hertert S., Hunninghake D.B., Probstfield J.L. (1997). Recruitment for controlled trials: literature summary and annotated bibliography. Control Clin Trials.

[2] McDonald A., Knight R., Campbell M.K. (2006). What influences recruitment to randomised controlled trials? A review of trials funded by two UK funding agencies. Trials.

[3] Prescott R.J., Counsell C.E., Gillespie W.J. (1999). Factors that limit the number, quality and progress of randomised controlled trials. Health Technol Assess.

[4] McCulloch P., Taylor I., Sasako M., Lovett B., Griffin D. (2002). Randomised trials in surgery: problems and possible solutions. BMJ.

[5] Stirrat G.M., Farrow S.C., Farndon J., Dwyer N. (1992). The challenge of evaluating surgical procedures. Ann R Coll Surg Engl.

[6] Wente M.N., Seiler C.M., Uhl W., Buchler M.W. (2003). Perspectives of evidence-based surgery. Dig Surg.

[7] Gordon B., Mackrodt C., Fern E. (1998). The Ipswich Childbirth Study: 1. A randomized evaluation of two stage postpartum perineal repair leaving the skin unsutured. Br J Obstet Gynaecol.

[8] Neumayer L., Giobbie-Hurder A., Jonasson O. (2004). Open mesh versus laparoscopic mesh repair of inguinal hernia. N Engl J Med.

[9] Anvari M., Allen C., Marshall J. (2006). A randomised controlled trial of laparoscopic nissen fundoplication versus proton pump inhibitors for treatment fo patients with chronic gastroesophageal reflux disease: one-year follow-up. Surg Innov.

[10] Howes N., Chagla L., Thorpe M., McCulloch P. (1997). Surgical practice is evidence based. Br J Surg.

[11] Rojavin M.A. (2005). Recruitment index as a measure of patient recruitment activity in clinical trials. Contemp Clin Trials.

[12] (2006). SPSS Base 11.0 for Windows user's guide.

[13] (2007). Stata Statistical Software: release 10.0.

[14] Haidich A., Ioannidis J. (2003). Late-starter sites in randomized controlled trials. J Clin Epidemiol.

[15] Frobell R.B., Lohmander L.S., Roos E.M. (2007). The challenge of recruiting patients with anterior cruciate ligament injury of the knee into a randomized trial comparing surgical and non-surgical treatment. Comtemp Clin Trials.

